# Effect of Cedar Honey in the Treatment of Oral Lichen Planus 

**Published:** 2014-07

**Authors:** Majid Sanatkhani, Pegah Mosannen Mozafari, Maryam Amirchaghmaghi, Mohsen Najafi Fathi, Mohammad Sanatkhani, Naghmeh Sarjami, Amir Abbas Azarian

**Affiliations:** 1 *Oral and Maxillofacial Diseases Research Center, School of Dentistry, Mashhad University of Medical Sciences, Mashhad, Iran.*; 2 *Department of Veterinary and Biotechnology, Razi Vaccine and Serum Institute, Mashhad,Iran.*; 3 *Undergraduate Student of Dentistry, Szeged Dental School, Szeged, Hungary.*; 4 *General Dentist, private clinic, Tehran, Iran.*; 5 *Department of Statistics, Payame Noor University, Tehran, Iran. *

**Keywords:** Atrophic, Cedar honey, Clinical trial, Erosive, Iran, Oral lichen planus, Pain, Treatment.

## Abstract

**Introduction:**

Oral Lichen Planus (OLP) is a chronic mucocutaneous disease with an immunological etiology. This study was conducted to evaluate the effect of cedar honey in the treatment of erosive- atrophic OLP.

**Materials and Methods:**

Thirty patients with a confirmed clinical and histopathologic diagnosis of OLP participated in this randomized clinical trial in Mashhad Dental School. Patients were randomly allocated into one of two groups. Both groups received standard OLP treatment (dexamethasone mouthwash 0.5 mg three times daily and fluconazole capsule 100 mg daily). The intervention group received cedar honey (20 ml three times daily, via a swish and swallow technique) in addition to standard treatment. The patients were followed for 4 weeks. The pain and severity of the lesions were recorded at the initial visit and follow ups. All recorded data were analyzed using the chi-square test, T-test, and analysis of variance (ANOVA) using SPSS version 11.5. A p-value less than 0.05 was considered significant.

**Results:**

Both groups had a marked reduction in pain, size of erosive area, and atrophic lesions, particularly in the first follow-up period, but there was no significant difference between the two groups (P>0.05). Honey was effective in the healing of ulcerative lesions (average recovery in the experimental group was 69% while the average relief of ulcerative lesion in the control group was 50%), but the difference was not significant (P=0.896).

**Conclusion:**

No significant difference was found in the treatment of atrophic and erosive lesions of OLP through use of honey as an alternative treatment. However, this approach may be effective in managing ulcerative lesions of OLP; although more research with a larger sample size is necessary.

## Introduction

Lichen Planus (LP) is a chronic, inflammatory mucocutaneous disorder that is thought to be the result of an autoimmune process. Oral Lichen Planus (OLP) is a common disorder with a reported prevalence of 1.27% and is most commonly seen in women aged between 30 and 60 years (1). Although oral lesions of OLP may be the only manifestation of the disease, the scalp, nails, and genitalia can also be affected. There is a premalignant potential for OLP according to some studies (2,3). The etiology of OLP is still unclear, but there is evidence that immunological processes have a key role. Cytotoxic cells are directed against basilar keratinocytes, which leads to degeneration and lyses of basal cells (4,5). Tumor necrosis factor α (TNFα), granulocyte-macrophage colony-stimulating factor (GM-CSF) and interleukin 6 (IL-6) cytokines are released, provoking a local inflammatory response. Furthermore, the equilibrium between oxidant/antioxidant is disturbed. Lipid peroxidative products are elevated and DNA damage within the epidermis is observed (2,6). OLP may present in two main types: a reticular type which requires no treatment or an atrophic/erosive type which does require therapy because of its tendency towards developing into a malignancy. 

Corticosteroids are the drugs of choice for the treatment of OLP. Other treatments such as cyclosporine extracorporeal photo-chemotherapy, tacrolimus and pimecrolimus have been studied (5); however the associated side effects and complications, especially in long-term administration, are disadvantageous. Therefore, the search for an alternative natural or herbal drug with anti-inflammatory properties has evolved; whether taken as monotherapy or in combination with systemic corticosteroids.

Honey is a potential alternative therapy, due to its antioxidant and anti-inflammatory properties, as well as its ability to inhibit the release of TNF-α, IL-1β, and IL-6 (7), free radicals and nitric oxide (8). This study was conducted to evaluate the complementary administration of Ziziphus- spina-Christi honey (known as cedar or conar honey in Persian and collected from the south of Iran) in addition to topical corticosteroids in treatment of erosive-atrophic OLP. This clinical trial was registered in clinical trial.gov and is identified as NCT01974414.

## Materials and Methods

*Participants*

This prospective randomized controlled clinical trial was performed in the Department of Oral and Maxillofacial Medicine of Mashhad Dental School, Mashhad, Iran from September 2011 to September 2012. Thirty-six patients were assessed according to eligibility requirements. As a pilot study, a sample size of 36 was calculated because this study was first to investigate honey and OLP. An electronic random number generator was used to create a list of random numbers (via URL: http://stattrek.com/statistics/random-number-generator.aspx) such that 18 patients were randomly allocated to each intervention and control group.

Inclusion criteria were: 

1) Clinically and histophatologically confirmed OLP without dysplasia in histo- pathologic evaluation Clinical diagnosis was based on interlacing white striped appearingbilaterally on the posterior buccal mucosa which was observed by two oral medicine specialists. Histopathlogic diagnosis was verified by two independent oral pathologists based on hydropic degeneration on the basal layer, band-like lymphocytic infiltration, saw shaped rete ridges and some degree of keratosis in keratinized lesions. 

2) Severity of pain≥2 (visual analog scale (VAS)> 3.5)

3) Severity of lesions≥2 (Thongprasom criteria)

4) Absence of any treatment in the last month

5) Absence of kidney or liver diseases (due to systemic administration of fluconazole to both groups)

Exclusion criteria were:

1) Evidence of lichenoid reaction in clinical or histopathologic assessment (due to specific etiologies [e.g. drugs such as cyclosporine, fluoroquinolone] or dental restoration)

2) Loss of follow up

3) Pregnant patients

4) Diabetic patients

5) Any other mucosal disease

6) Any severe systemic disease

7) Patients who refuse doctor's advice.

Although rare, if any unexpected adverse effect of honey was observed, the trial was stopped in all enrolled patients. 

*Interventions, randomization and blinding*

The two study groups were provided with standard treatment for OLP (Mouthwash, dexamethasone [Iran Hormoon Inc, Iran] 0.5 mg QID and fluconazole capsule (Zahravi Inc, Iran] 100 mg daily). 

Fluconazole capsules were used as an anti-fungal drug, so all patients were checked for blood urea nitrogen (BUN), creatinine, serum glutamic pyruvic transaminase (SGPT), and serum glutamic oxaloacetic transaminase (SGOT) levels to exclude patients with any liver and kidney problem before initiation of trial. The intervention group (A) received cedar honey, 20 ml three times daily via the swish and swallow technique in addition to the standard treatment, while the control group (B) received standard treatment only. Due to the lack of any similar study we used a treatment protocol proposed by Motallebnejad to study the effect of honey on oral mucositis (20 ml honey, three times a day for via the swish and swallow technique) (9). Provision of the cedar honey was from a secure source; a company specializing in honey products from Kohgilooyeh and the Boyer Ahmad province. The honey sample was initially tested for purity and natural and biological factors such as enzyme activities (amylase, oxidase, peroxidase, catalase and invertase) and total polyphenols with standard flavonoid antioxidant compounds. Also, the same honey was tested for anti-bacterial activity and physiochemical properties. After quality control, the honey was applied clinically as described above.

Patients were told they might or might not receive honey treatment and were educated how to consume the honey. This trial was conducted in accordance with ethical principles and was approved by the ethics committee of Mashhad University of Medical Science. All patients signed an informed consent form before initiation of research. 

At the patient’s first visit, information including age, gender, disease process, medical history, family history, and clinical signs and symptoms were documented. All patients had a histopathologic record confirming OLP, but the records were reviewed by an expert oral pathologist to exclude lichenoid reaction. 

Determination of whether a patient should be in group A (intervention group) or group B (control group) was made by reference to a statistical series based on random number of calculation. 

In this study, we had no honey placebo because of associated side effects, particularly on the teeth. All the patients in the intervention group were instructed to use honey for 4 weeks. A weekly follow up was planned for all patients. A flow diagram of the trial is shown in Figure 1. All patients received standard treatment for OLP.

**Fig 1:**
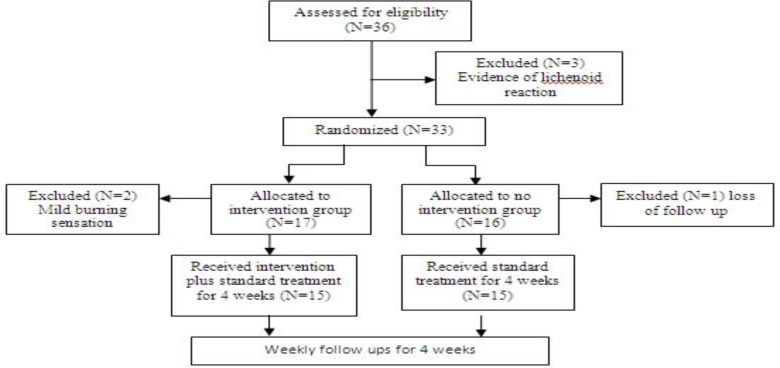
Study flow diagram

*Clinical* *assessment*

Several clinical indexes were included such as VAS, pain index (PI), severity index (SI), size of atrophic lesion (at. size), size of erosive lesion (er. size) and also

improvement in these indexes. All the values for clinical indexes were assessed and recorded at the start and the end of each week by two researchers (PMM and MS) who were blind to the study group. A sterile caulis was used to measure the maximum diameter of erosive and atrophic lesions, and the maximum width perpendicular to the maximum diameter was recorded as at. size or er. size. Grading of size was defined as: 0=Normal mucosa, 1= 0–1 cm lesion size, 2 = 1–3 cm lesion size, 3= more than 3 cm lesion size. SI was calculated as follows (10):

SI=Σ (score of erosive lesion × Grade of size of erosive lesion) +Σ (score of atrophic lesion × Grade of size of atrophic lesion). The score of erosive lesion and atrophic lesion were 2 and 1.5, respectively.

Pain or burning sensation was assessed using VAS.

Patients marked the point from 0 (no pain) to 10 (extreme pain), represent- ting their present pain perception. PI was defined as 0= no pain, 1= mild pain (0<VAS<3.5),

2= moderate pain (3.5< VAS<7), 3= severe pain (7<VAS<10). 

Improvement of lesions and symptoms were calculated by these formulas: 

Improvement of severity index =$\frac{\mathrm{SI in first visit}-\mathrm{SI in last visit}}{\mathrm{SI in first visit}}$

Improvement of pain index= $\frac{\mathrm{PI in first visit}-\mathrm{PI in last visit}}{\mathrm{PI in first visit}}$

Grading of improvement in SI and PI is shown in Tables. 1 and 2. Improvement of lesions was calculated in five grades for each study index (PI, SI, atrophic size, erosive size) as follows: 

Complete improvement=100%;considerable improvement=75–100%;moderate improvement=25–75%;slight improvement= 0–25%; deterioration ≤0%.

*Adverse reactions*

In case of an adverse reaction, close observation was performed. In case of serious reactions, treatment was discontinued and the subject was sent for treatment to an outside clinic not involved in the research.

*Follow-up assessment*

All patients were scheduled for a 4-week follow-up assessment. Patients with complete eradication of the erosion at any time were followed up for 1 month to detect recurrences. If the SI and PI were 0, the treatment was stopped. Patients who still had erosions after 1 month of treatment were referred for other therapies including topical, intralesional or systemic corticosteroids and topical immunos-uppressant or laser therapy. 

*Statistical analysis*

Statistical analysis was performed with SPSS 11.5 for Windows (SPSS Inc, Chicago). The differences in erosive size and VAS scores between the beginning and the end of the treatment in each group were calculated by ANOVA tests. The normality of the variable was assessed with the chi-square test. The differences in erosive size and VAS scores between the two groups were analyzed by T-test and ANOVA tests. All statistical tests were performed using a significance level of P<0.05 (two tailed). The statistician was blinded to the groups and intervention. 

## Results

Thirty-six patients were assessed according to eligibility requirements. Three patients were excluded because there was some evidence of lichenoid reaction in the histopathologic and clinical evaluation. Seventeen patients entered the intervention group but two were excluded due to a mild burning sensation, following honey consumption. This was an unexpected complication which was not reported previously in the literature and, because of ethical issues in human research, these patients were excluded. These patients continued standard treatment and underwent follow up in the oral medicine department. Sixteen patients entered the control group, but one of them was excluded because of an inability to participate in follow-up appointments. Finally 30 patients, including two men (6.66%) and 28 women (93.33%) aged 18 to 75 years, were included in the trial; 15 in the intervention group and 15 in the control group. The mean age of the patients was 46.7±31.9 years [intervention group: F= 46.8±8.9, control group: F= 45.3± 8.9, M=54±15]. All patients received dexa- methasone mouthwash and fluconazole capsule as the standard OLP treatment. 

There were no differences between the two groups in age, gender, erosive size and VAS scores, severity of lesions and previous treatment for LP at the start of treatment(P>0.05).The baseline comparison of the two groups is shown in (Table. 1). The site of lesions is shown in (Table. 2).

**Table 1:** Baseline characteristics of the study participants

**Variable **	**Intervention group** **N=15**	**Control group** **N=15**	**P-Value**
Age(mean ± SD)	46.8 ±8.9	46.53±10.75	0.10
Gender, n (%) Male Female	0 15(100%)	2(13.33) 13(86.66)	0.09
Previous treatment, (%) No treatment Treated before	4(26.7%) 11(73.3%)	8(53.3%) 7(46.7%)	0.13
VAS score	4.66	4.98	0.06
Pain index (mean ± SD)	2.20	2.33	0.12
Severity index	3.53	3.67	0.87
Atrophic size area (mm^2^)	1352	2110	0.16
Erosive size area (mm^2^)	76.9	71.5	0.77

* Significant difference between two groups

*Outcome and estimation*

Data from 30 patients, 15 in the intervention group and 15 in control group, were analyzed.

*Pain of lesions*

Pain of lesions was significantly reduced in both groups at the end of study (P<0.001), but there were no differences between the two groups (P=0.775). When considering time intervals, maximum pain reduction was achieved in the first follow up in both groups. Fig. 2 illustrates pain reduction in study groups. Pain reduction in the first follow up was significantly greater than that in the second (P=0.01) and third (P=0.001) follow ups, although both groups were similar in pain reduction (P=0.969). Improvement of clinical indexes is shown in (Table. 3). Pain improvement was more obvious in the first follow up compared with other follow-up seasons (P=0.01).

**Table 2:** Involvement of different sites in patients (intervention group=15, control group=15

**Site of lesions**	**Intervention group N (%)**	**Control group N (%)**	**Total N (%)**	**P. value**
Buccal mucosal	14 (93.3%)	14 (93.3%)	28 (93.3%)	0.31
Tongue	9 (60%)	12 (80%)	21 (70%)	0.84
Maxillary gingiva	7 (46.6%)	6 (40%)	13 (43%)	0.15
Mandibular gingiva	8 (53.3%)	10 (66.6%)	18 (60%)	0.93
Upper labial mucosa	1 (6.6%)	4 (26.6%)	5 (16.6%)	0.01*
Lower labial mucosa	0 (0%)	2 (13.3%)	2 (13.3%)	0.0
Other site	0 (0%)	0 (0%)	0 (0%)	0.0

* Significant difference between two groups

**Table 3:** Improvement of clinical indexes in study groups

**Clinical Index in study groups**	**Complete Improvement (100%)**	**Considerable Improvement (75–100%)**	**Moderate Improvement (25–75%)**	**Slight Improvement ** **(0–25%)**	**Deterioration (0%)**
Pain and burning sensation					
Int.gr. Cont.gr. Total	9 9 18	0 1 1	4 3 7	2 2 4	0 0 0
Atrophic Size					
Int.gr. Cont.gr. Total	0 0 0	9 7 16	5 5 10	1 1 2	0 2 2
Erosive Size					
Int.gr. Cont.gr. Total	4 1 5	0 0 0	2 4 6	8 6 14	0 0 0
Severity Index					
Int.gr. Cont.gr. Total	0 0 0	0 0 0	6 3 9	9 12 21	0 0 0

* Int gr.= Intervention Group, Cont G.= Control Group

**Fig 1:**
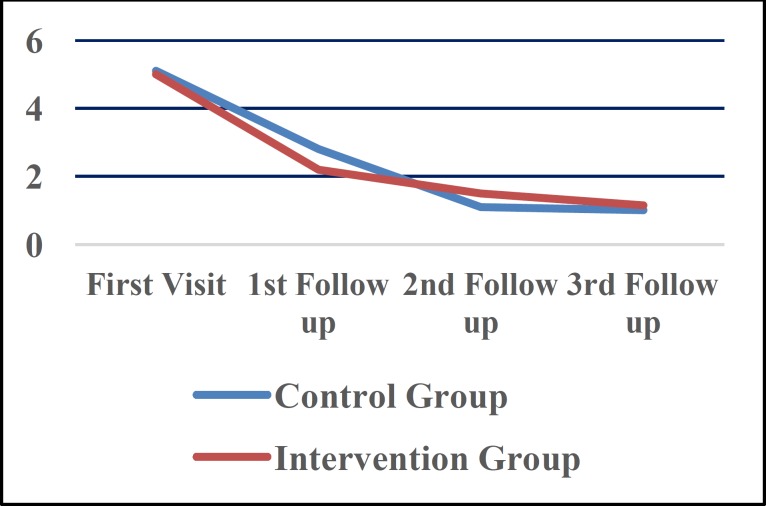
Pattern of pain reduction in study groups

*Size of erosive lesions*

**Fig 3:**
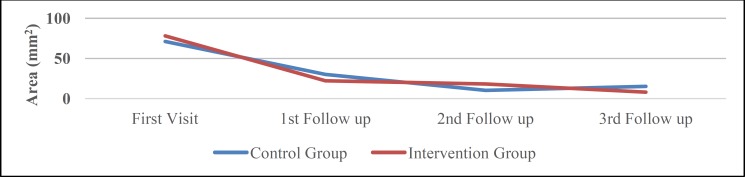
Erosive Size Reduction in study groups

The size of erosive lesions was reduced in both groups, but the difference was not significant (P=0.133 for the control and P=0.231 for the intervention group). Although this reduction was greater in the intervention group, there was no difference between the two groups (P=0.85) (Fig. 3). Reduction in size was greater in the first follow up in the intervention group than other follow-up seasons.

*Size of atrophic lesions*

Atrophic size was significantly decreased in both groups (P=0.017 for the control group and P=0.014 for the intervention group). However, atrophic size reduction was more prevalent in the intervention group (P=0.0451).

*Severity of lesions*

SI was recorded according to the Thongprasom criteria. SI was significantly decreased in both groups, but the groups were similar in severity index and severity improvements (P=0.859). Table. 3 shows SI and severity improvements in different follow-up seasons. The average improvement of erosive lesions in the intervention group was 69%, while the average recovery in control group was 50% in erosive lesions. Such effect was not observed in atrophic lesions. In other words, the effect of honey on ulcerative lesions is greater than that on atrophic lesions, but the difference was not statistically significant (P=0.896).

*Improvement of size of lesions in different sites of involvement*

**Fig 4:**
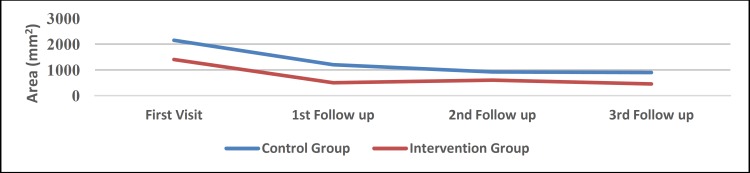
Atrophic Size Reduction in study groups

The size of the atrophic lesion in the buccal mucosa and tongue were significantly reduced in both groups (P<0.05), but the size of atrophic lesions in maxillary and mandibular gingiva and labial mucosa was not significantly decreased. In other words, it seems that lesions of the gingiva and labial mucosa are refractory to treatment. Table .4 shows the mean improvement in the different sites of involvement in study groups. Fig. 4 shows atrophic size reduction in the study groups.

**Table 4:** Mean improvement in different sites of involvement in study groups

Location	Improvement In Intervention Group (%)	Improvement In Control Group (%)	P-Value
Buccal mucosa	49	55	0.80
Tongue	56	43	0.95
Upper lip mucosa	0	61	0.86
Upper keratinized gingiva	49	9	0.65
Lower keratinize gingiva	37	50	0.96

*Safety analysis*

Two patients reported a mild burning sensation due to honey consumption, and were excluded from the study. This complication was resolved after treatment cessation.

*Follow-up analysis*

Since atrophic lesions were not fully recovered, patients remained under observation until complete resolution of lesions. However, honey was not administered and only standard treatment was continued.

## Discussion

Recently, use of natural drugs, such as honey, has gained considerable interest. Although honey is very sweet, it has some components with antioxidant properties which makes it a useful substance for the treatment of OLP. Notably, antioxidising components are flavonoids and polyphenols, enzymes (glucose oxidase, catalase), organic acids, ascorbic acid, carotenoid-like substances, amino acids, and proteins (11). Honey has a number of antiproliferative properties as well. Jaganathan et al. showed that honey can induce apoptosis in human colon cancer cells by arresting the cells at the subG1 phase. Honey rich in phenolic and tryptophan was more effective in inhibiting the proliferation of cancer cells (12). 

OLP is a chronic autoimmune disease, treated with standard therapies including topical or systemic corticosteroids. Management of OLP can be challenging as these treatments can have significant side effects when used on a long-term basis or if repeated short courses are needed for control. There is a need for safe and effective anti-inflammatory medications for OLP used as the sole treatment or in conjunction with corticosteroids (13,14).

Inflammation plays an important role in OLP pathogenesis. Inflammatory factors such as nuclear factor kappa B (NF-κB) are produced during the inflammation. Honey is also rich in polyphenols, which are strong anti-inflammatory substances (9,15). Honey is also a strong anti-bacterial substance, which promotes wound healing processes (16,17), as well as ulcerative lesions in OLP. However, because of the small sample size of ulcerative lesions (13 patients of 30), the results were not significant.

In our study, because the honey was not available in jelly form, patients kept the honey in their mouth for one minute before swallowing it to avoid it being washed out by saliva and because of the dental risks associated with long contact with honey. The purpose of this study was to evaluate the efficacy of cedar honey for the treatment of erosive OLP. Both the intervention and control groups received topical dexamethasone and capsule fluconazole as the standard treatment .The results showed that there were no significant differences between the two groups in the reduction of clinical signs (SI) and the alleviation of the clinical symptoms (pain and burning sensation). Both groups had a significant reduction in SI and PI over 4 weeks, but no significant differences between the two groups were observed (in other words, intragroup analysis showed significant differences in each group but inter group analysis did not show any significant differences).

In this study, cedar honey was chosen, because of its high flavonoid and enzyme content (including amylase, oxidase, catalase, and peroxidase) and because it is considered to be the best choice among different honey types (18,19).

To date, no other similar studies investigating the role of honey in the treatment of OLP have been published, and we are therefore not able to compare our results with any other research. Topical royal jelly (a honey bee product) has been used to treat recurrent aphthous stomatitis and the results showed that royal jelly was effective in achieving a complete improvement of lesions in 100% of patients over 6 months of follow up (20).

Alternative natural drugs such as curcuminoids, and Aloe Vera (AV) have been used previously in OLP; but due to differences in research design and drug mechanism, we cannot compare our results with these studies (21,22).

Reddy et al evaluated the effectiveness of (AV) gel in the treatment of OLP when compared with triamcinolone acetonide and concluded that AV gel can be considered a safe alternative treatment for OLP (23). Furthermore, Mansurian et al researched the therapeutic effects of AV mouthwash with triamcinolone acetonide 0.1% (TA) on OLP and concluded that AV mouthwash is an effective substitute for TA in the treatment of OLP (24).

In contrast, Salazar-Sánchez et al evaluated the efficacy of the topical application of AV in OLP compared with placebo and revealed no statistically significant differences between the two groups in relation to pain after 6 and 12 weeks (25).

Saawarn and associates worked on a study to evaluate the effects of oral lycopene on the atrophy and erosions of OLP. Thirty patients were divided among a placebo and intervention group. All patients receive 8 mg of lycopene and continued in the study for 8 weeks. At the end of the study, all patients in the intervention group experienced recovery of greater than 50%, but only 10 patients in placebo group had a recovery of 50% or more (14).

Chainani-Wu N studied the efficacy of curcuminoids in controlling the signs and symptoms of OLP at doses of 6,000 mg/d (three divided doses), as well as the safety of this dose. They concluded that curcuminoids at doses of 6,000 mg/d in three divided doses are well tolerated and may prove efficacious in controlling signs and symptoms of OLP (21).

Mousaviet al evaluated the effectiveness of Ignatia homeopathic remedy at 30 °C in the management of OLP. In this single-blind randomized controlled clinical trial, 30 consecutive patients with oral lesions clinically and histologically consistent with erosive and/or atrophic OLP were recruited. Results suggest that Ignatia has a beneficial effect in the treatment of OLP in selected patients (26). Taheri studied the efficacy of the Elaeagnus angustifolia (a plant with anti-inflammatory and analgesic qualities) in treating OLP. Twenty-eight patients divided among an Elaeagnus angustifolia gel group and a placebo group used the gel of the plant three times a day for 14 days. It was reported that patients in the intervention group experienced greater recovery than those in the placebo group (27).

In the majority of studies described, placebo group patients did not receive corticosteroids; however, in our study topical corticosteroids were administered for ethical consideration. Furthermore, in this study the limited number of patients as well as the short-term treatment and follow-up periods might be responsible for the similar treatment efficacy and absence of any adverse reactions in the two groups.

Other factors such as psychological etiologies must be considered in future studies. In one study Delavarian showed that psychological stressors can aggravate OLP, and psychological treatment can be significantly effective in treatment of OLP (28). We did not find any significant differences in treatment outcomes between the two groups. We did not assess psychologic status in our study sample; although it is interesting to speculate whether we would have found a significant difference if we had assessed the two groups before treatment. It seems that intervention group had more stressors (e.g. divorce and other problems) and more psychological problems (e.g. depression, anxiety) than the control group, and this could be responsible for the similar treatment outcomes in the two groups. We suggest psychological assessment in future research to balance study participants in the two groups. Furthermore, most of the employed patients were allocated to the intervention group by chance, and disuse of honey is probable. 

In our research no benefit was found for administration of cedar honey. This may be due to: 1) small sample size, 2) presence of psychological stress, 3) absence long-term contact the lesions with the honey, 4) short-term follow up, or 5) loss of compliance. Therefore, it is strongly recommended that more research with a larger sample size, longer follow up and controlled psychological factors be conducted.

## Conclusion

Over a period of short-term administration, the efficacy of topical honey was greater in a number of clinical aspects. The drug was safe with no adverse effects. However, more research with larger sample size is necessary for a full evaluation of the efficacy of honey.
